# Pan-Cancer Analysis of the Characteristics of LY96 in Prognosis and Immunotherapy Across Human Cancer

**DOI:** 10.3389/fmolb.2022.837393

**Published:** 2022-05-11

**Authors:** Kechao Nie, Jing Li, Luqi Peng, Mei Zhang, Wei Huang

**Affiliations:** ^1^ Department of Integrated Traditional Chinese and Western Internal Medicine, The Second Xiangya Hospital, Central South University, Changsha, China; ^2^ Department of Integrated Traditional Chinese and Western Internal Medicine, Xiangya Hospital, Central South University, Changsha, China

**Keywords:** lymphocyte antigen 96, tumor microenvironment, tumor immunity, prognosis, immunotherapy

## Abstract

Lymphocyte antigen 96 (LY96) is implicated in tumorigenesis by modulating host immunity. However, an integrated pan-cancer analysis of LY96 in prognosis and immunotherapy across human cancers is still lacking. Therefore, we analyzed the LY96 expression and its prognostic role in tumors by multiple databases. We also investigated the correlation between LY96 and copy number, DNA methylation, somatic mutation, microsatellite instability (MSI), tumor mutation burden (TMB), tumor microenvironment (TME), and immune cell infiltration across human cancers. In addition, the biological processes related to LY96 across various tumors and the correlation between LY96 and 50% inhibitive concentration (IC50) of various drugs were investigated. We found that LY96 was differently expressed between tumor and normal tissues and was significantly upregulated in most types of cancers. LY96 was gradually upregulated from stages I to IV in several cancers. Moreover, we found LY96 may play a prognostic role in most cancers, and patients with high or low LY96 expression often show different clinical outcomes. LY96 was also associated with copy number, DNA methylation, somatic mutation, MSI, TMB, TME characteristics, and immune cell infiltration in cancers. LY96 may also regulate classic tumor-associated pathways in several cancers and is related to drug resistance. This article may help to elucidate the role of LY96 in tumorigenesis, which may promote the development of immunotherapy and targeted therapy in cancers.

## Introduction

In recent years, cancer immunotherapy (CIT) has made rapid progress in the treatment of various types of cancers effectively through the interaction between the human immune system and cancer (Ribas and Wolchok, 2018). Different types of immunotherapies, including immune checkpoint therapy, CAR T cell therapy, cancer vaccine, and tumor neoantigen therapy, have already been applied in the clinic (Yang, 2015). Although immunotherapy has been applied to most tumors, its ultimate efficacy is still limited, and even some patients may not respond to CIT (Ju et al., 2021). In addition, immunotherapy can cause a variety of side effects, including intolerance and death in patients (Pardoll, 2012). Therefore, there is an urgent need to mine a predictive biomarker to assess the response to these immunotherapy approaches to determine early patient benefits. Previous studies found that CIT responses were highly related to specific indicators such as tumor mutation burden (TMB), DNA methylation, somatic mutation, microsatellite instability (MSI), and copy number variation (CNV). Therefore, it is urgent to find an accurate biomarker to predict the clinical outcome and CIT responses in tumors.

Lymphocyte antigen 96 (LY96), also known as myeloid differentiation 2 (MD2), is required for the activation of TLR4 by lipopolysaccharide (LPS), which plays an important role in innate immunity and is the first line of defense against microbial infection (Dou et al., 2013). LY96 plays a vital role in inflammation-related and immune-related diseases such as Crohn’s disease (Yang et al., 2021), rheumatoid arthritis (Rychkov et al., 2021), and inflammatory diabetic cardiomyopathy (Wang et al., 2020a). Recently, several studies revealed that LY96 was tightly correlated with tumor initiation and progression. LY96 interacted with TLR4 and activated the nuclear factor-κB (NF-κB) pathway, and thereby promoted the production of pro-inflammatory cytokines and adhesive molecules in colon cancer cells, which accelerate colon cancer growth and lung metastasis (Rajamanickam et al., 2020). LY96, markedly expressed in gastric cancer cells, can activate macrophage-mediated NF-κB and STAT3 pathways, thereby promoting tumor progression (Zhou et al., 2018).

The role of LY96 in several specific tumors was well investigated; however, there is no pan-cancer analysis of LY96 in various types of cancers. Therefore, we analyzed the LY96 expression and its prognostic role in tumors by multiple databases, including Genotype Tissue-Expression (GTEx), Cancer Cell Line Encyclopedia (CCLE), TCGA, and cBioPortal. We also investigated the correlation between LY96 and copy number, DNA methylation, somatic mutation, MSI, TMB, tumor microenvironment (TME), and immune cell infiltration in various types of cancers. In addition, we also analyzed the biological processes related to LY96 across various tumors and found a correlation between LY96 and the 50% inhibitive concentration (IC50) of various drugs. Our study revealed that LY96 can be used as a prognostic indicator in various tumors and was highly correlated with different clinical indicators (copy number, DNA methylation, somatic mutation, MSI, and TMB), TME and immune cell infiltration, and IC50 of various drugs. This study may promote the understanding of LY96 and tumor immunity in pan-cancer.

## Materials and Methods

### Analysis of LY96 Expression in Different Tumor Tissues and Cells

The expression profiles and clinical data in human cancers of TCGA and GTEx were obtained from the UCSC XENA website (https://xenabrowser.net/datapages). The TCGA database included 33 types of cancer tissues and adjacent samples, whereas the GTEx database included normal samples from 31 different types of tissues (Supplementary Table S1). The expression data of 30 types of different tumor cell lines were downloaded from the CCLE database (https://portals.broadinstitute.org/ccle/).

Data extraction and collation were conducted by Strawberry Perl (version 5.32.0). Expression data were log2-transformed, and two sets of *t*-tests were conducted between cancer and adjacent samples. A *p* value less than 0.05 was considered statistically significant. Data analyses were performed by R software (version 3.6.1).

### Analysis of the Correlation Between LY96 Expression and TNM Stage and Prognosis

We analyzed the correlation between LY96 expression and TNM stage using data from the UCSC XENA database. Then, the UALCAN database (http://ualcan.path.uab.edu/) was used to analyze the correlation between LY96 expression and age and gender across human cancers. Furthermore, different prognostic indexes, including overall survival (OS), disease-specific survival (DSS), disease-free interval (DFI), and progression-free interval (PFI), were selected to study the relationship between LY96 expression and patient prognosis. The Kaplan–Meier survival method and Cox log test were used to determine prognosis. A *p*-value less than 0.05 was considered statistically significant. The packages “Survminer” and “survival” were used in R software.

### Analysis of the Correlation Between LY96 and Copy Number, DNA Methylation, Somatic Mutation, Microsatellite Instability, and Tumor Mutation Burden

The cBioportal database was used to explore the mutation and amplification of LY96 in 33 types of cancers. The Pearson’s correlation between gene expression and copy number was calculated in each tumor. In each tumor, the correlation between gene expression and methylation of the gene promoter was calculated by Pearson correlation. Generally, hypermethylation of the promoter leads to downregulation of mRNA expression. Methylation data were derived from cBioportal (www.cbioportal.org), and HM450 data were used. We also analyzed the difference in mutation profiles in patients with high and low LY96 expression. In addition, the correlation between LY96 expression and MSI and TMB was analyzed across cancers. Packages “RColorBrewer” and “reshape2” in R software were used.

### LY96 and Tumor Immune Microenvironment

LY96 expression and immunotherapeutic relevant signatures were analyzed using the previously published methods, including EMT, immune checkpoint, and CD8 T effector. Furthermore, we calculated immune and stromal scores of each patient with cancer using the ESTIMATE algorithm, which reflects the immune characteristics of tumors to some degree. In addition, the TIMER2 database was used to investigate the correlation between LY96 expression and different types of immune infiltrated cells in most cancers. We also investigated whether LY96 was related to immune infiltrated cells across 33 cancers using the CIBERSORT algorithm. The packages “estimate,” “ggpubr,” “ggExtra,” and “ggplot2” were used in R.

### Biological Processes Related to LY96

Gene Set Enrichment Analysis (GSEA) and Gene Set Variation Analysis (GSVA) were performed to detect the biological processes related to LY96 in various types of cancers. The top 300 genes highly associated with LY96 were selected from a gene set and performed GSEA analysis using “clusterprofiler” packages in R. In terms of GSVA, the sample was divided into two high-expression and low-expression groups using the median of GSVA score (LIMMA package). In each tumor, the top 15 pathways positively and negatively correlated with LY96 were plotted in cancers.

### Correlation Between LY96 and IC50 of Different Types of Drugs

The relationship between the gene and the IC50 of 198 types of drugs was analyzed using the GDSC2 database (https://www.cancerrxgene.org/). The IC50 differences of each drug in the high- and low-LY96 expression groups were plotted using cell line expression profile data in GDSC2.

### Statistical Analysis

All gene expression profiles were log2-transformed in this study. A *t* test was used to compare the differences in LY96 expression in normal and tumor tissues across various types of cancers. A log-rank test (*p* <0.05) in Kaplan–Meier survival was used to identify the survival difference between the high- and low-LY96 expression groups. A *p*-value less than 0.05 was regarded as significant in Cox regression analysis. The correlation between LY96 and various variables was analyzed using Spearman’s or Pearson’s test. *p* < 0.05 was considered significant.

## Results

### LY96 Expression in Different Tumor Tissues and Cells

**FIGURE 1:**
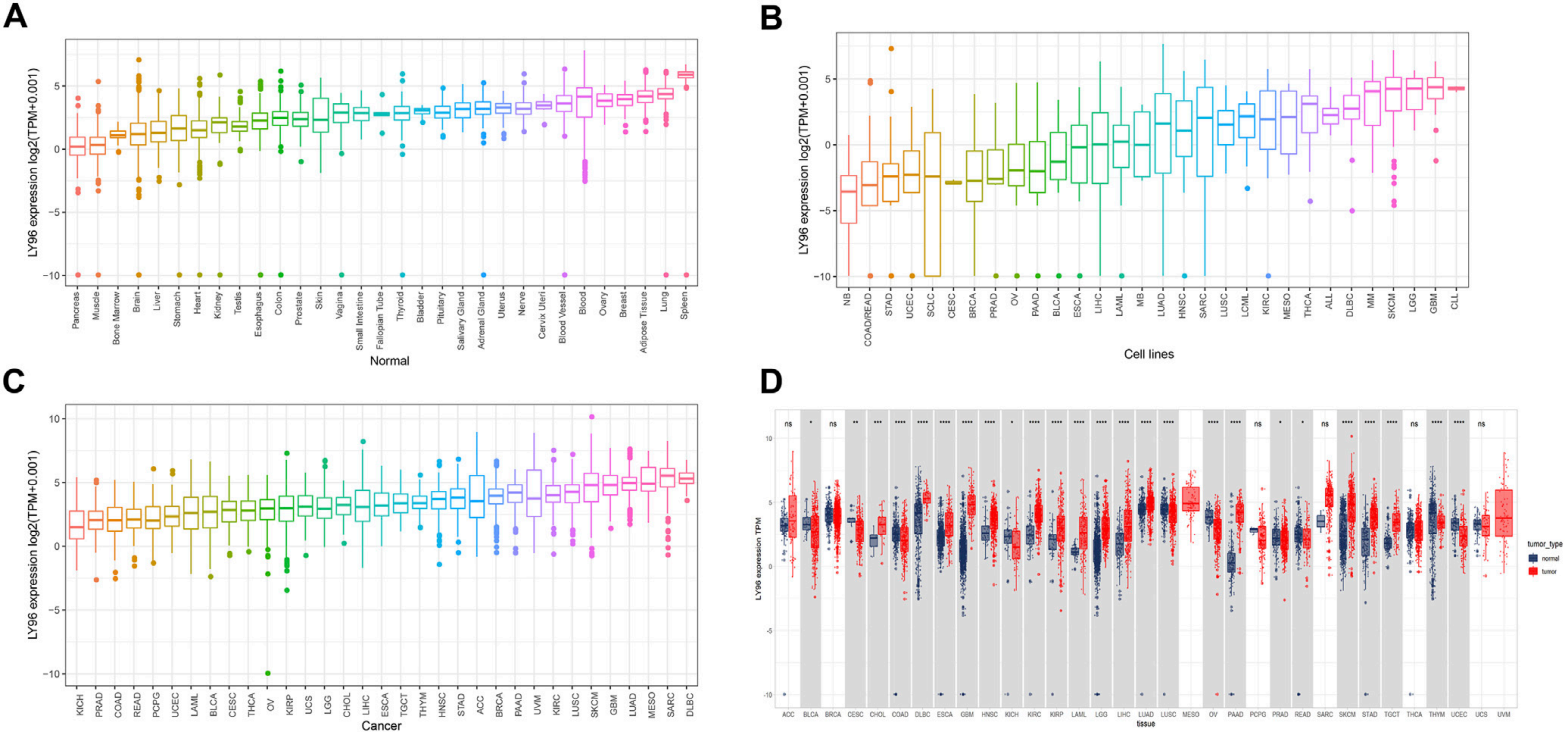
Expression characteristic of LY96. **(A–C)** Expression of LY96 in normal tissues **(A)**, cell lines **(B)**, and cancers **(C)**. **(D)** Comparison of LY96 expression in cancer and adjacent tissues.

We analyzed the LY96 expression in normal tissues using GTEx databases and found LY96 showed higher expressed in the breast, adipose, lung, and spleen tissues, whereas LY96 showed lower expression in the pancreas, muscle, bone marrow, and brain tissues (Figure 1A). We also analyzed the LY96 expression in different tumor cell lines in the CCLE database, and the results revealed that LY96 was highly expressed in SKCM, LGG, GBM, and CLL, and was low expressed in NB, COAD/READ, STAD, and UCEC cells (Figure 1B). Among 33 types of tumor tissues, high LY96 expression was found in KICH, PRAD, COAD, and READ, and low expression of LY96 was found in LUAD, MESO, SARC, and DLBC tissues (Figure 1C). In addition, compared with adjacent tissues, LY96 expression was significantly different in 22 types of tumor tissues (Figure 1D). Concretely, compared with normal tissues, LY96 was significantly upregulated in CHOL, DLBC, ESCA, GBM, HNSC, KIRC, KIRP, LAML, LGG, LIHC, LUAD, PAAD, SKCM, and TGCT. However, higher expression of LY96 relative to normal tissues was detected in BLCA, CESC, COAD, KICH, LUSC, OV, PRAD, READ, THYM, and UCEC. Intriguingly, the largest expression changes between tumor and normal tissues were detected in GBM and SKCM.

### LY96 Was Associated With TNM Stage

**FIGURE 2:**
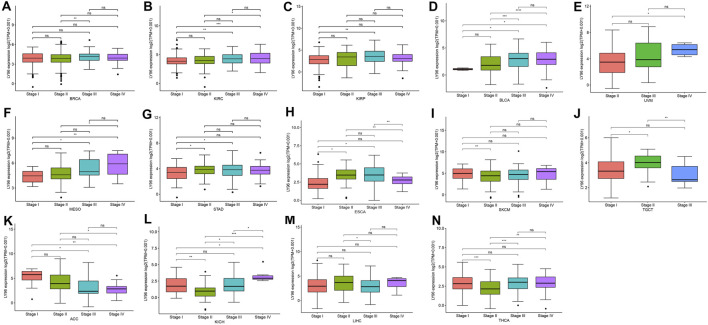
Correlation between LY96 expression and stage in **(A)** breast invasive carcinoma (BRCA), **(B)** kidney renal clear cell carcinoma (KIRC), **(C)** kidney renal papillary cell carcinoma (KIRP), **(D)** bladder urothelial carcinoma (BLCA), **(E)** uveal melanoma (UVM), **(F)** mesothelioma (MESO), **(G)** stomach adenocarcinoma (STAD), **(H)** esophageal carcinoma (ESCA), **(I)** skin cutaneous melanoma (SKCM), **(J)** testicular germ cell tumors (TGCT), **(K)** adrenocortical carcinoma (ACC), **(L)** kidney chromophobe (KICH), **(M)** liver hepatocellular carcinoma (LIHC), and **(N)** thyroid carcinoma (THCA).

We analyzed the correlation between LY96 expression and TNM stage and the results revealed that LY96 was significantly associated with clinical stage in BRCA, KIRC, KIRP, BLCA, UVM, MESO, STAD, ESCA, SKCM, TGCT, ACC, KICH, LIHC and THCA (Figures 2A–O). LY96 expression was gradually upregulated from TNM I to TNM IV stages in BRCA, KIRC, KIRP, BLCA, UVM, MESO, and STAD (Figures 2A–G). In contrast, LY96 expression was gradually downregulated from TNM I to TNM IV stages in ACC (Figure 2K). Higher LY96 expression was found in II and III stages, whereas lower expression of LY96 was found in I and IV stages in ESCA and SKCM (Figures 2H,I). Lower LY96 expression was found in II and III stages, whereas higher expression of LY96 was found in I and IV stages in KICH and THCA (Figures 2L,N). Furthermore, we found LY96 was significantly associated with age in CESC, KIRP, PAAD, READ, SARC, STAD, THYM, THCA, UCS, and UCEC (Supplementary Figure S1). LY96 expression was gradually downregulated with age in CESC, KIRP, PAAD, READ, STAD, THCA, and UCS (Supplementary Figures S1A–D, S1F, S1H, S1I). LY96 was also significantly associated with gender in KIRP, KIRC, and READ (Supplementary Figure S2). Concretely, female patients showed higher LY96 levels than male patients in KIRP and KIRC (Supplementary Figures S2A, S2B), whereas female patients showed lower LY96 levels than male patients in READ (Supplementary Figure S2C).

### Correlation Between LY96 and Prognosis

**FIGURE 3:**
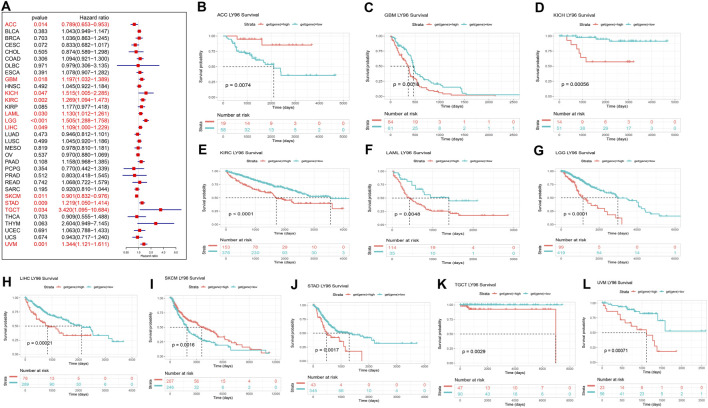
Correlation between LY96 expression and overall survival (OS). **(A)** Forest plot of LY96 expression and OS across cancers. Kaplan–Meier analysis of LY96 expression and OS in **(B)** adrenocortical carcinoma (ACC), **(C)** glioblastoma multiforme (GBM), **(D)** kidney chromophobe (KICH), **(E)** kidney renal clear cell carcinoma (KIRC), **(F)** acute myeloid leukemia (LAML), **(G)** brain lower grade glioma (LGG), **(H)** liver hepatocellular carcinoma (LIHC), **(I)** skin cutaneous melanoma (SKCM), **(J)** stomach adenocarcinoma (STAD), **(K)** testicular germ cell tumors (TGCT), and **(L)** uveal melanoma (UVM).

The prognostic role of LY96 expression across cancers was analyzed using data downloaded from the TCGA database. The Cox proportional analysis showed that LY96 expression was significantly correlated with OS in ACC, GBM, KICH, KIRC, LAML, LGG, LIHC, SKCM, STAD, TGCT, UVM (Figure 3A). Except in ACC and SKCM (HR <1), LY96 was a risky gene in GBM, KICH, KIRC, LAML, LGG, LIHC, STAD, TGCT, and UVM (HR >1). Kaplan–Meier survival analysis showed that patients with high LY96 expression exhibited longer OS than patients with low LY96 expression in ACC and SKCM (Figures 3B,I). However, patients with high LY96 expression exhibited shorter OS in GBM, KICH, KIRC, LAML, LGG, LIHC, STAD, TGCT, and UVM (Figures 3C–H, J–L).

**FIGURE 4:**
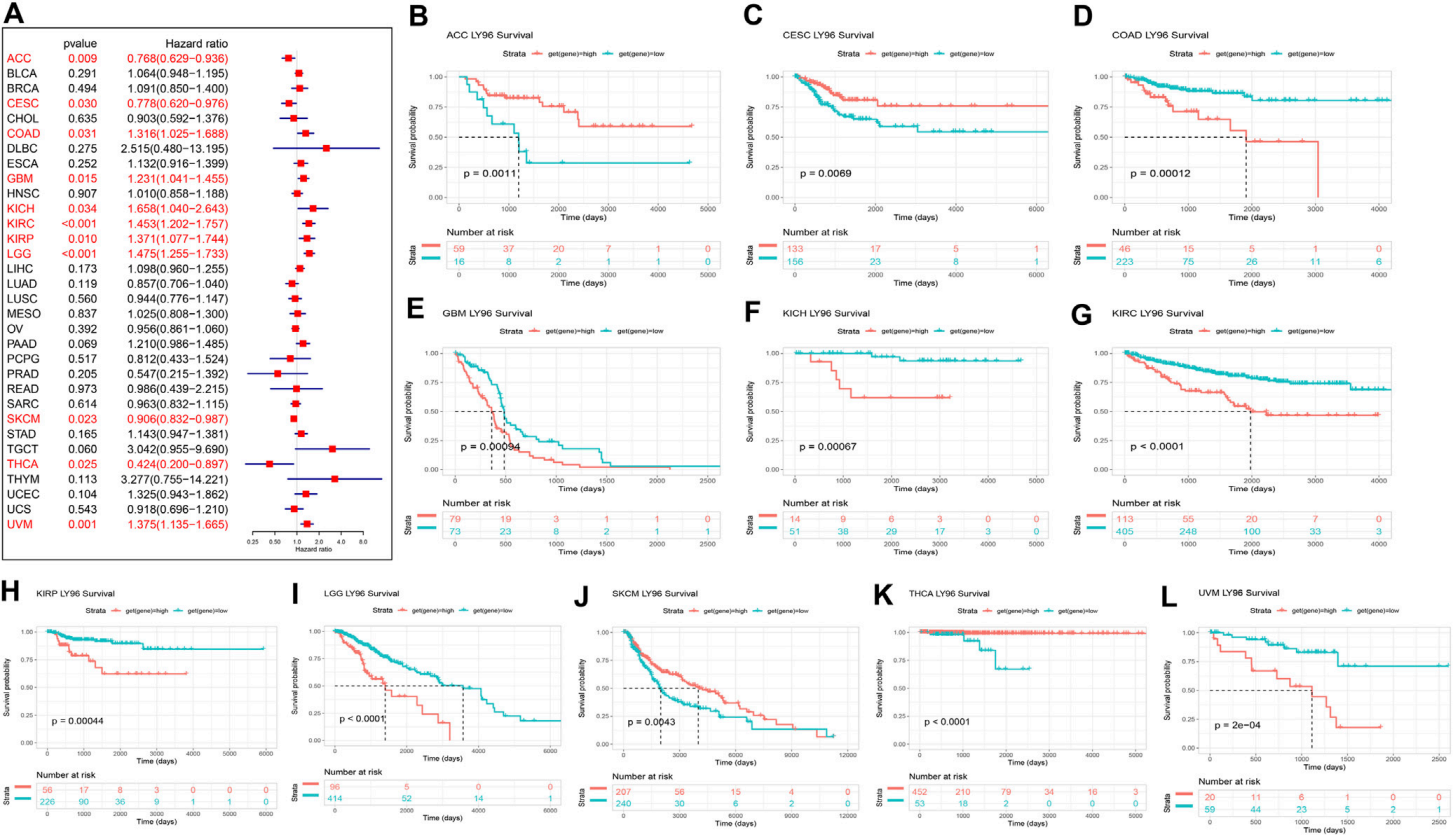
Correlation between LY96 expression and disease-specific survival (DSS). **(A)** Forest plot of LY96 expression and DSS across cancers. Kaplan–Meier analysis of LY96 expression and DSS in **(B)** adrenocortical carcinoma (ACC), **(C)** cervical squamous cell carcinoma, endocervical adenocarcinoma (CESC), **(D)** colon adenocarcinoma (COAD), **(E)** glioblastoma multiforme (GBM), **(F)** kidney chromophobe (KICH), **(G)** kidney renal clear cell carcinoma (KIRC), **(H)** kidney renal papillary cell carcinoma (KIRP), **(I)** brain lower grade glioma (LGG), **(J)** skin cutaneous melanoma (SKCM), **(K)** thyroid carcinoma (THCA), and **(L)** uveal melanoma (UVM).

Furthermore, Cox proportional analysis showed LY96 expression was significantly correlated with DSS in ACC, CESC, COAD, GBM, KICH, KIRC, KIRP, LGG, SKCM, THCA, and UVM (Figure 4A). LY96 was a protective gene in ACC, CESC, SKCM, and THCA (HR <1), and was identified as a risky gene in COAD, GBM, KICH, KIRC, KIRP, LGG, and UVM (HR >1). KM analysis revealed that patient with high LY96 expression exhibited longer DSS than patients with low LY96 expression in ACC, CESC, SKCM, and THCA (Figures 4A,B,J,K), however, patients with high LY96 expression exhibited shorter DSS in COAD, GBM, KICH, KIRC, KIRP, LGG, and UVM (Figures 4C–I, L).

LY96 expression was correlated with DFI only in KIRP, and KM analysis revealed that patients with high LY96 expression exhibited a shorter DFI than patients with low LY96 expression (Supplementary Figures S3A, S3B). In terms of the PFI indicator, Cox proportional analysis showed LY96 expression was significantly correlated with PFI in ACC, CESC, CHOL, GBM, KICH, KIRC, KIRP, LGG, PRAD, THYM, and UVM (Supplementary Figure S3C). KM analysis revealed that patients with high LY96 expression exhibited longer PFI than patients with low LY96 expression in ACC, CESC, and CHOL (Supplementary Figures S3D, S3F). However, patients with high LY96 expression exhibited shorter PFI in GBM, KICH, KIRC, KIRP, LGG, PRAD, THYM, and UVM (Supplementary Figures S3G–S3N).

### Correlation Between LY96 and Copy Number, DNA Methylation, Somatic Mutation, Microsatellite Instability, and Tumor Mutation Burden

**FIGURE 5:**
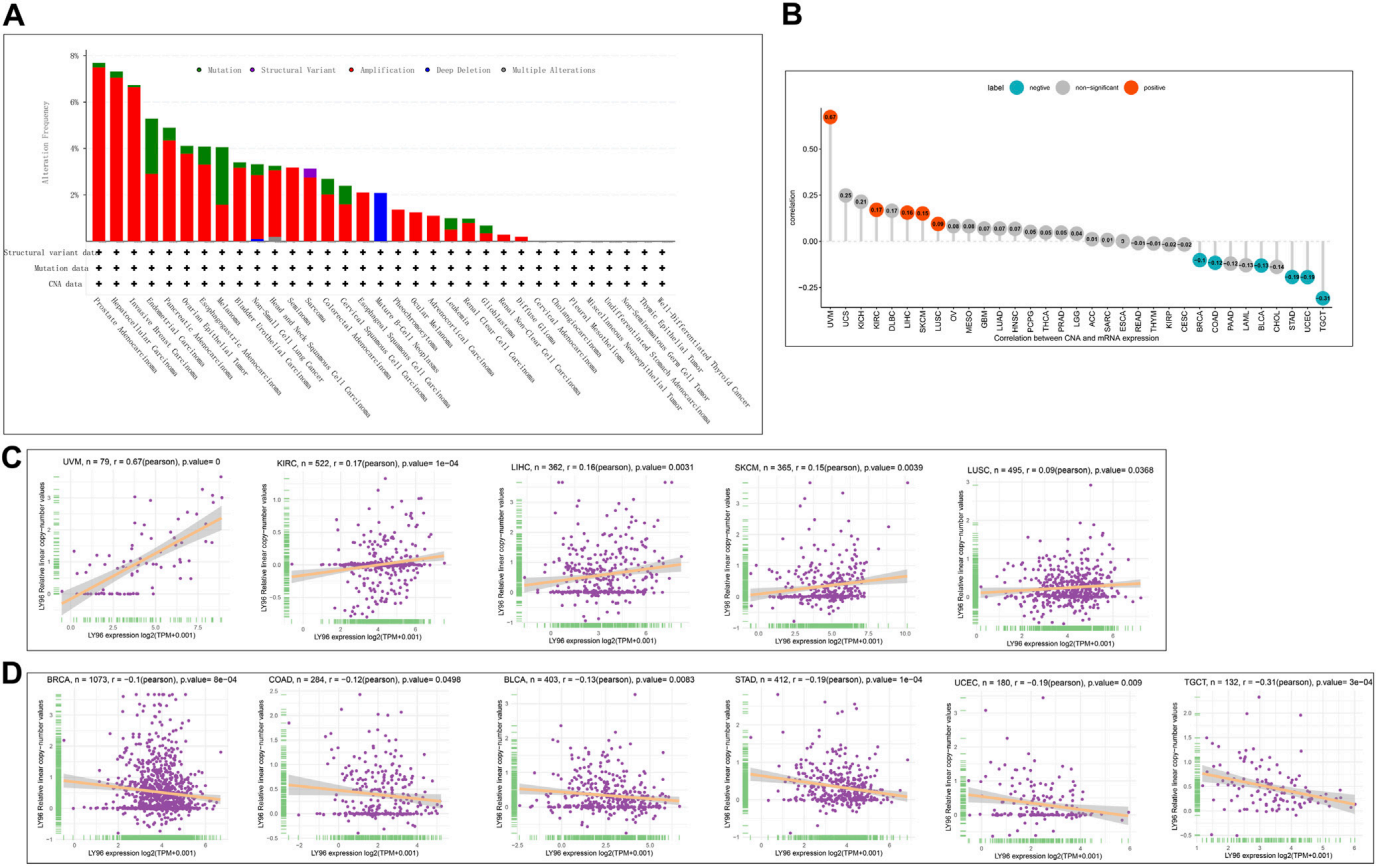
Correlation between LY96 expression and copy number variation (CNV). **(A)** CNV of LY96 across cancers. **(B)** Correlation between LY96 expression and CNV. **(C)** LY96 expression was positively correlated with CNV in uveal melanoma (UVM), kidney renal clear cell carcinoma (KIRC), liver hepatocellular carcinoma (LIHC), skin cutaneous melanoma (SKCM), and lung squamous cell carcinoma (LUSC). **(D)** LY96 expression was negatively correlated with CNV in testicular germ cell tumors (TGCT), uterine corpus endometrial carcinoma (UCEC), stomach adenocarcinoma (STAD), bladder urothelial carcinoma (BLCA), colon adenocarcinoma (COAD), and breast invasive carcinoma (BRCA).

**FIGURE 6:**
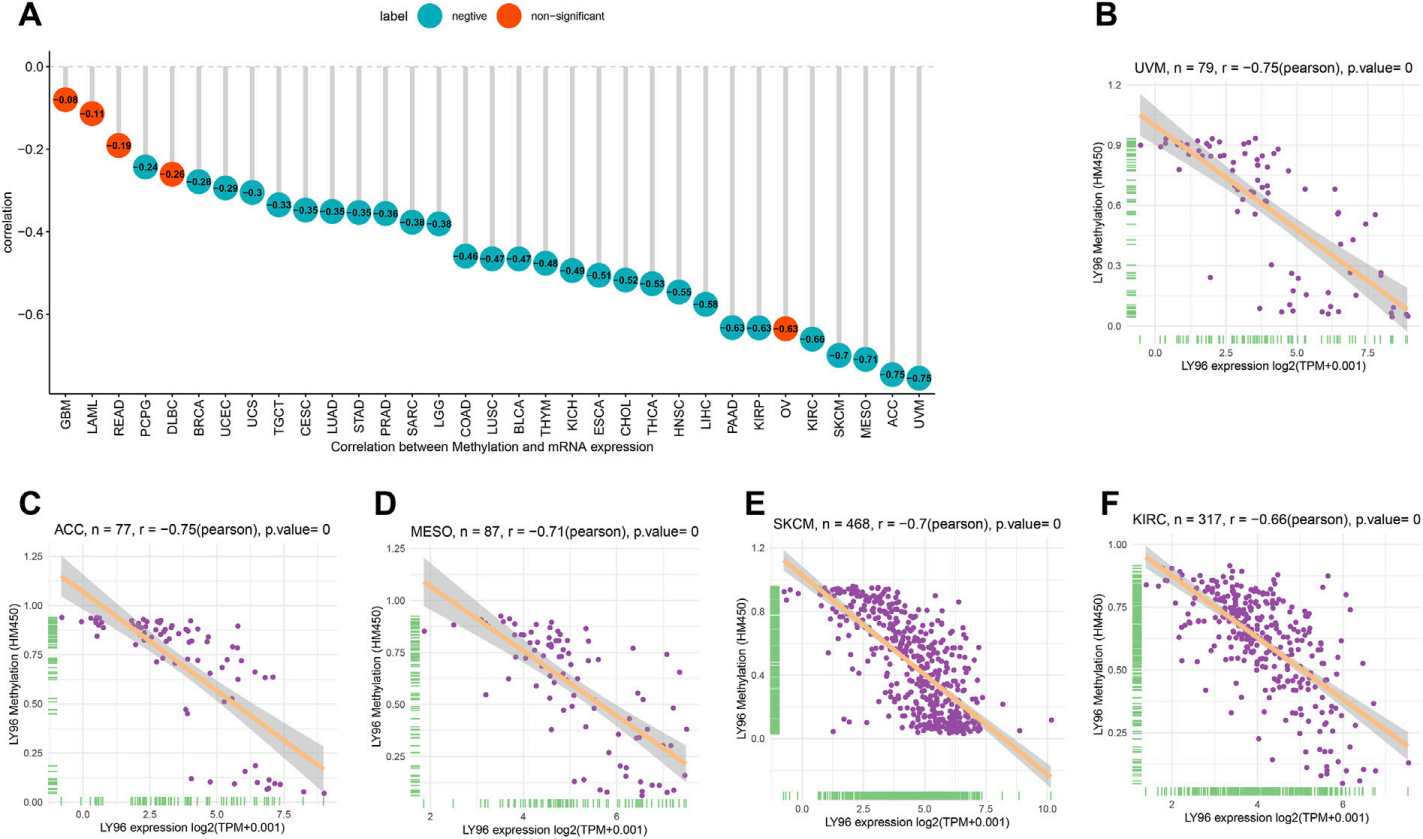
Correlation between LY96 expression and DNA methylation. **(A)** Correlation between LY96 expression and DNA methylation across cancers. **(B–F)** Correlation between LY96 expression and DNA methylation in **(B)** uveal melanoma (UVM), **(C)** adrenocortical carcinoma (ACC), **(D)** mesothelioma (MESO), **(E)** skin cutaneous melanoma (SKCM), and **(F)** kidney renal clear cell carcinoma (KIRC).

Subsequently, we explored the correlations between LY96 expression and copy number, DNA methylation, somatic mutation, MSI, and TMB. The cBioportal database was used to reveal the mutation of LY96 in cancers, and results indicated that the highest alteration frequency occurred in prostate adenocarcinoma, hepatocellular carcinoma, and invasive breast carcinoma, whereas LY96 showed the lowest alteration frequency in well-differentiated thyroid cancer, thymic epithelial tumor, and non-seminomatous germ cell tumor (Figure 5A). Amplification was the main type of variation of LY96 among most cancers (Figure 5A). Among 33 types of cancer, LY96 expression was positively correlated with copy number variation (CNV) in UVM, KIRC, LIHC, SKCM, and LUSC (Figures 5B,C), whereas there was a negative correlation between LY96 expression and CNV in TGCT, UCEC, STAD, BLCA, COAD, and BRCA (Figures 5B,D). For DNA methylation, LY96 expression was negatively correlated with LY96 methylation in most cancers of 33 types of cancers (Figure 6A), and the highest correlation occurred in UVM, ACC, MESO, SKCM, and KIRC (Figures 6B–F).

**FIGURE 7:**
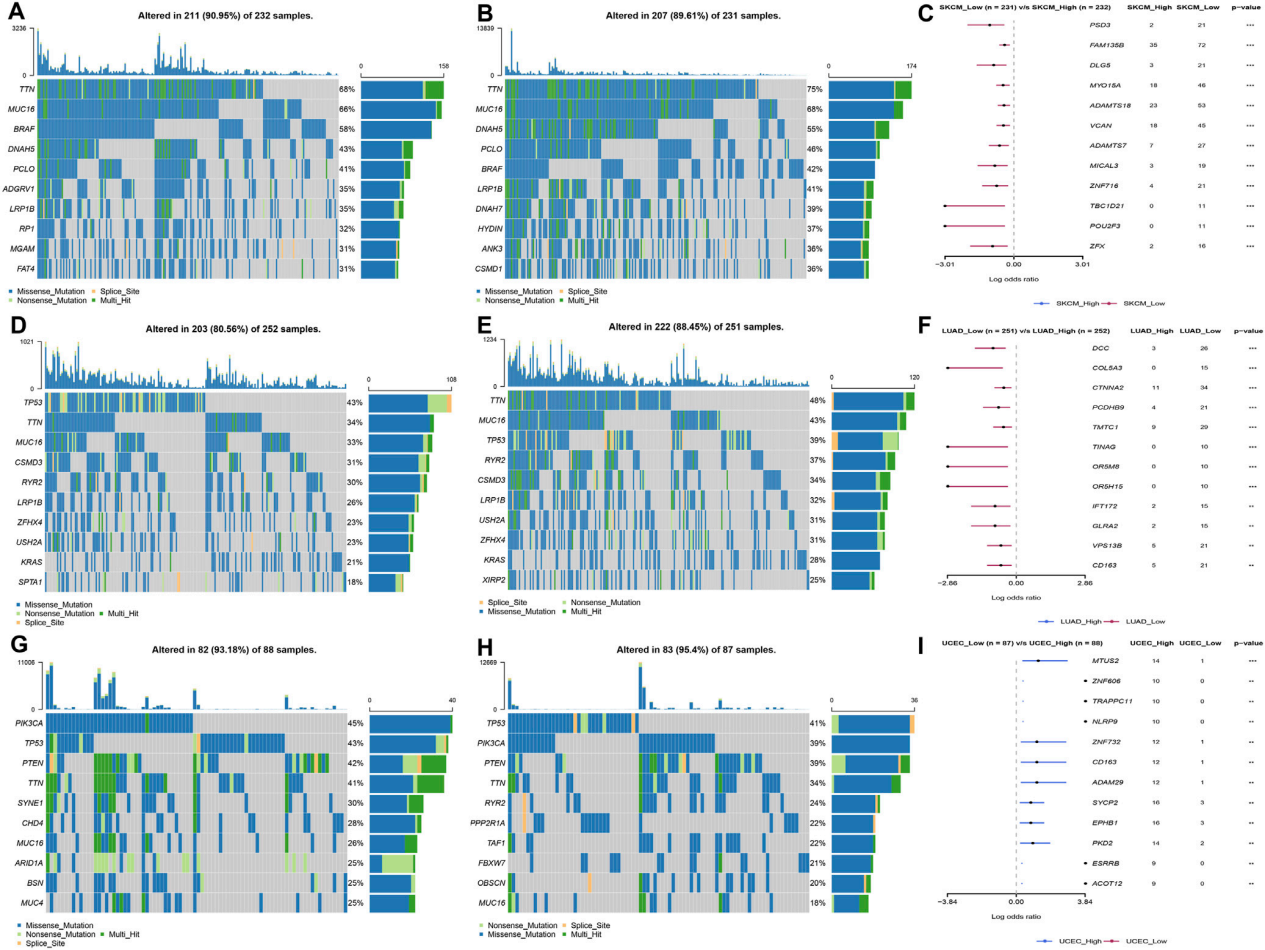
Somatic mutated profile between high- and low-LY96 expression groups. **(A–C)** Somatic mutated profile in high-LY96 expression group **(A)** and low-LY96 expression group **(B)** and differently mutated genes between high- and low-LY96 expression groups in **(C)** skin cutaneous melanoma (SKCM). **(D–F)** Somatic mutated profile in high-LY96 expression group **(D)** and low-LY96 expression group **(E)** and differently mutated genes between high- and low-LY96 expression groups in **(F)** lung adenocarcinoma (LUAD). **(G–I)** Somatic mutated profile in high-LY96 expression group **(G)** and low-LY96 expression group **(H)** and differently mutated genes between high- and low-LY96 expression groups in **(I)** uterine corpus endometrial carcinoma (UCEC).

**FIGURE 8:**
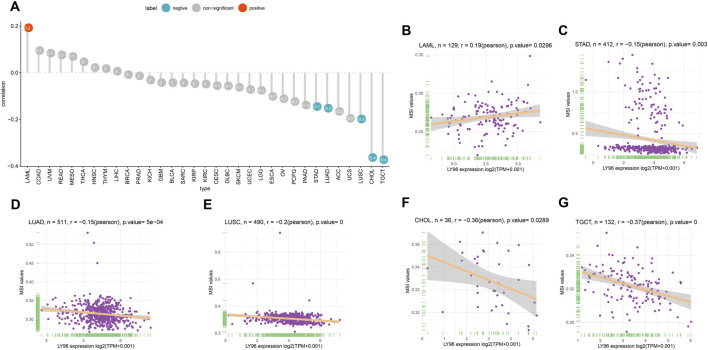
Correlation between LY96 expression and MSI. **(A)** Correlation between LY96 expression and MSI across cancers. **(B–G)** Correlation between LY96 expression and MSI in **(B)** acute myeloid leukemia (LAML), **(C)** stomach adenocarcinoma (STAD), **(D)** lung adenocarcinoma (LUAD), **(E)** lung squamous cell carcinoma (LUSC), **(F)** cholangiocarcinoma (CHOL), and **(G)** testicular germ cell tumors (TGCT).

**FIGURE 9:**
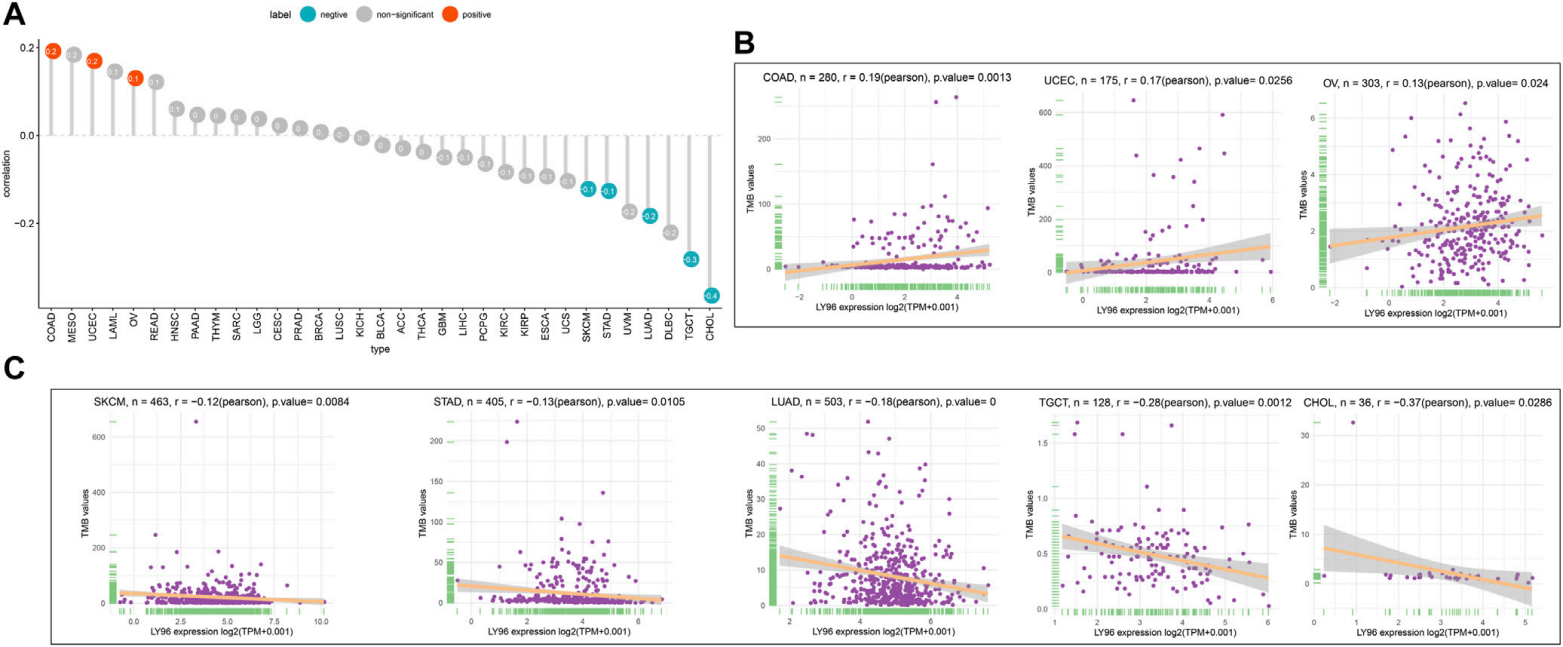
Correlation between LY96 expression and TMB. **(A)** Correlation between LY96 expression and TMB across cancers. **(B–C)** Correlation between LY96 expression and TMB in COAD, UCEC, and OV **(B)**. Correlation between LY96 expression and TMB in SKCM, STAD, LUAD, TGCT, and CHOL **(C)**.

The patients were divided into high- and low-expression groups according to the median of LY96 expression. We analyzed the differently mutated genes between the high- and low-expression groups, and the results showed the top mutations occurred in SKCM (Figures 7A–C), LUAD (Figures 7D–F) and UCEC (Figures 7G–I). Intriguingly, TTN, TP53, and MUC16 were the most commonly mutated genes. In terms of MSI, LY96 expression was positively correlated with MSI in LAML (Figures 8A,B), however, LY96 expression was negatively correlated with TGCT, CHOL, LUSC, LUAD, and STAD (Figures 8C–G). For TMB, LY96 was positively correlated with TMB in COAD, UCEC, and OV (Figures 9A,B) and was negatively correlated with TMB in CHOL, TGCT, LUAD, STAD, and SKCM (Figure 9C).

### Correlation Between LY96 and Tumor Microenvironment

**FIGURE 10:**
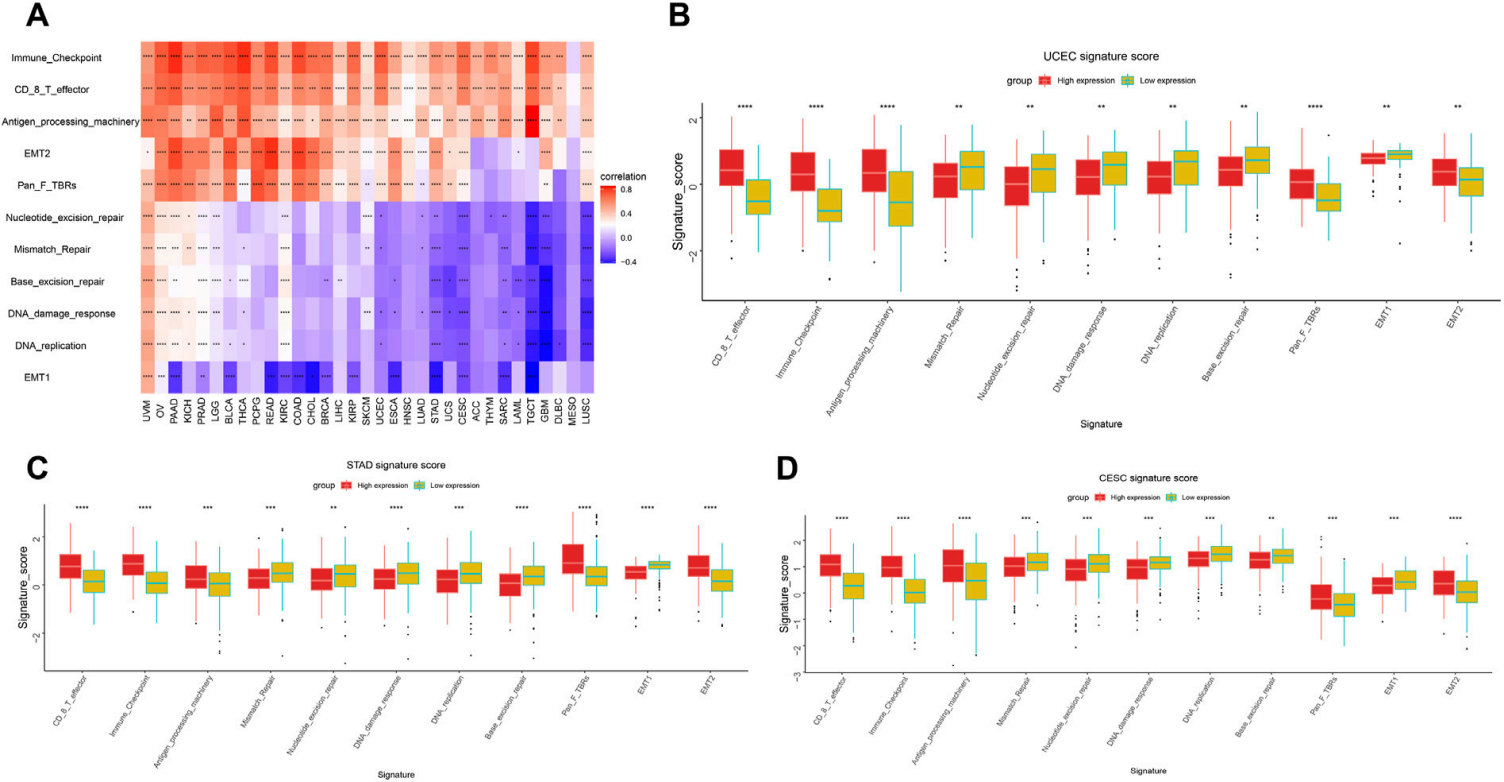
Correlation between LY96 expression and tumor microenvironment. Correlation between LY96 expression and immune-related pathways **(A)**. **(B–D)** Different signature score between high and low LY96 expression in **(B)** uterine corpus endometrial carcinoma (UCEC), **(C)** stomach adenocarcinoma STAD, and **(D)** cervical squamous cell carcinoma and endocervical adenocarcinoma (CESC).

**FIGURE 11:**
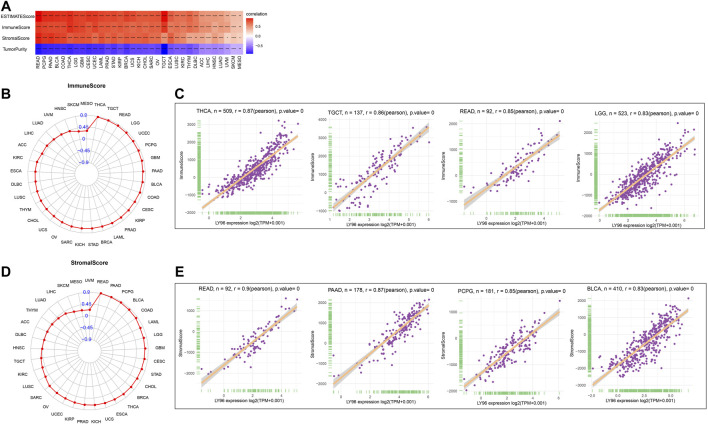
Correlation between LY96 expression and ESTIMATE score. **(A)** Correlation between LY96 expression and ESTIMATE score across cancers. **(B)** Correlation between LY96 expression and immune score across cancers. **(C)** Top 4 correlated cancers in terms of LY96 expression and immune score. **(D)** Correlation between LY96 expression and stromal score across cancers. **(E)** Top 4 correlated cancers in terms of LY96 expression and stromal score.

Increasing evidence shows that the tumor microenvironment plays a significant role in tumor initiation and progression. We analyzed the correlation between LY96 expression and immunotherapeutic relevant signatures, and the results indicated that LY96 was positively correlated with immune checkpoint, CD8 T effector, antigen processing machinery, EMT2, and PanF TBR’s pathway in most cancers (Figure 10A). In addition, LY96 was correlated with almost all pathways in UCEC, STAD, and CESC (Figure 10A), and different signature scores were obtained among patients with high and low LY96 expression (Figures 10B–D). LY96 was positively correlated with immune, stromal, and estimate scores and was negatively correlated with tumor purity across 33 cancers using the ESTIMATE algorithm (Figure 11A). In terms of immune score, a higher association was found between LY 96 and immune score in THCA, TGCT, READ, and LGG (Figures 11B,C). Similarly, LY96 was tightly correlated with stromal in READ, PAAD, PCPG, and BLCA (Figures 11D,E).

### Correlation Between LY96 and Immune Cell Infiltration

**FIGURE 12:**
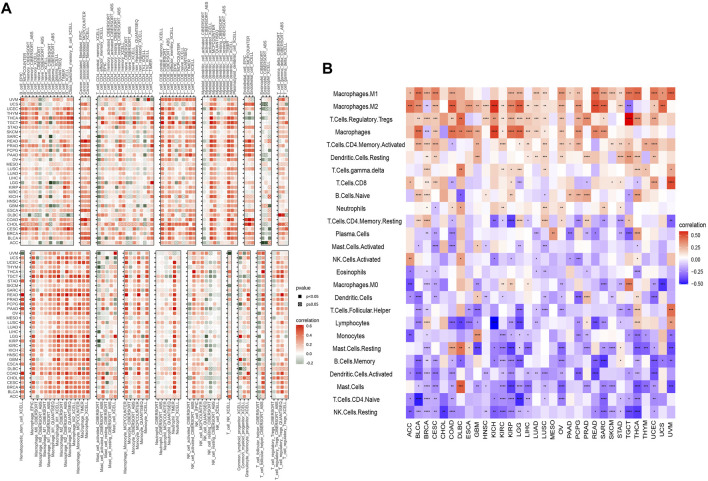
Correlation between LY96 expression and immune infiltrated cells. **(A,B)** Correlation between LY96 expression and immune infiltrated cells using TIMER2 data **(A)** and the CIBERSORT algorithm **(B)**.

We analyzed the correlation between LY96 expression and various types of immune cells in the TIMER2 database and the results demonstrated that LY96 expression was significantly correlated with most types of immune infiltrated cells in most cancers (Figure 12A). Furthermore, we also analyzed the correlation between LY96 and 24 types of immune infiltrated cells across cancers using the CIBERSORT algorithm. The results demonstrated that LY96 significantly correlated with immune cells in most cancers (Figure 12B). A higher correlation between LY96 and immune infiltrated cells occurred in BRCA (N = 23), THCA (N = 20), CESC (N = 18), KIRP (N = 18), and COAD (N = 17) (Supplementary Table S2). LY96 was significantly correlated with M1 macrophages and regulatory T cells (Tregs) in all aforementioned cancers and was negatively correlated with memory B cells, activated dendritic cells, mast cells, naive CD4 T cells,and resting NK cells. Furthermore, LY96 was significantly correlated with M2 macrophages, and macrophages in BRCA, KIRP, and COAD were negatively correlated with M2 macrophages and macrophages in CESC. In terms of various types of T lymphocytes, LY96 was positively correlated with activated CD4 memory T cells in BRCA and CESC and was negatively correlated with activated CD4 memory T cells in COAD. LY96 was positively correlated with CD8 T cells in BRCA, CESC, and KIRP and was negatively correlated with CD8 T cells in COAD and THCA.

We also analyzed whether the LY96 expression was correlated with immune-related gene set across cancers, and the results indicated that LY96 was significantly related to these gene set in most types of cancers (Supplementary Figure S4). In addition, LY96 was also highly related to most genes in autophagy, ferroptosis, M6A, and pyroptosis pathways (Supplementary Figure S5), which play a vital role in many biological processes.

### Gene Set Enrichment Analysis and Gene Set Variation Analysis

**FIGURE 13:**
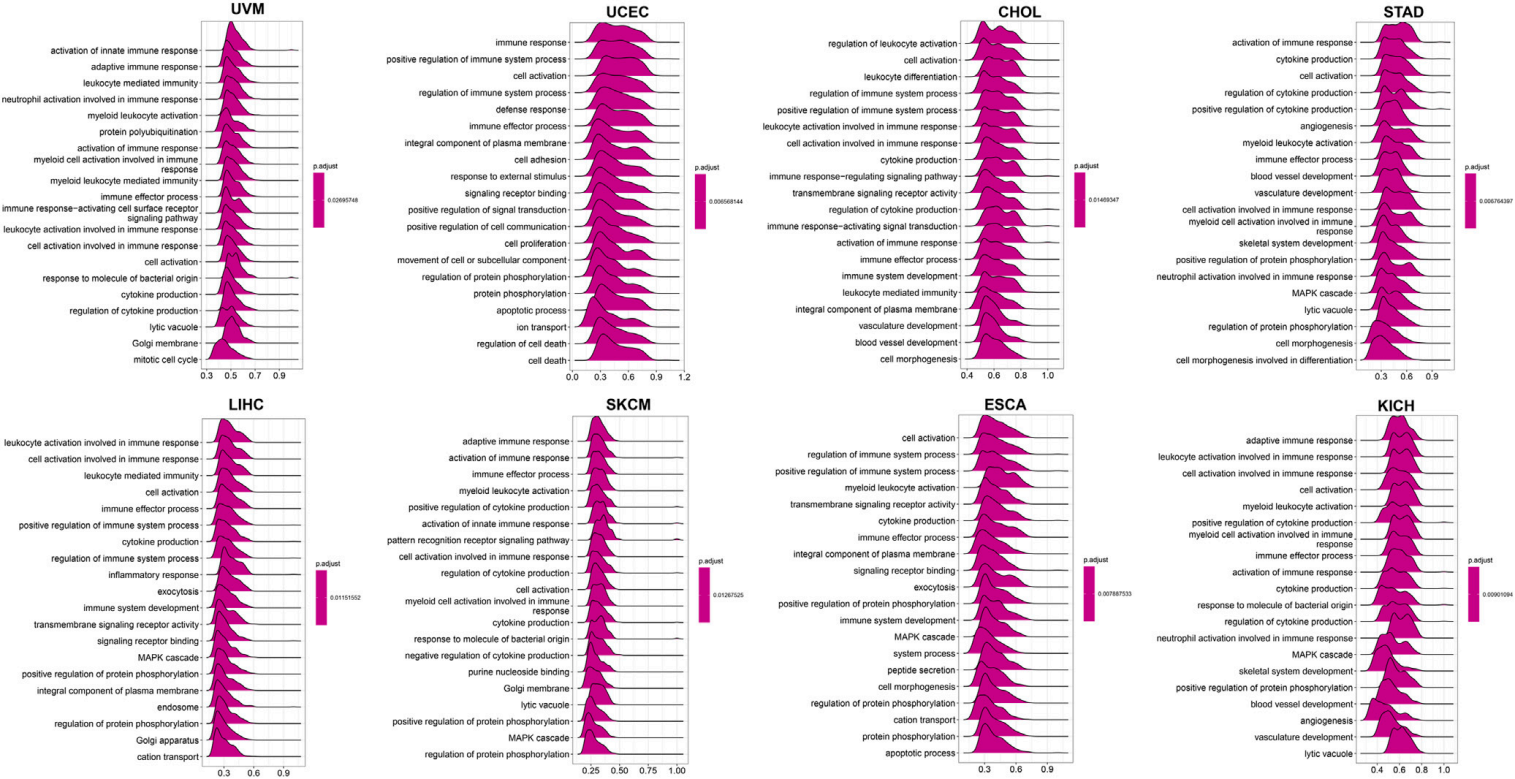
GO annotations related to LY96 expression in uveal melanoma (UVM), uterine corpus endometrial carcinoma (UCEC), cholangiocarcinoma (CHOL), stomach adenocarcinoma (STAD), liver hepatocellular carcinoma (LIHC), skin cutaneous melanoma (SKCM), esophageal carcinoma (ESCA), and kidney chromophobe (KICH).

To further explore the biological processes related to LY96 in various cancers, we conducted GSVA and GSEA analyses using R software. The results of GO annotation revealed that LY96 was significantly correlated with immune-related pathways, such as activation of innate immune response, adaptive immune response, immune response, and immune effector process in UVM, UCEC, CHOL, STAD, LIHC, SKCM, ESCA, and KICH (Figure 13). The KEGG pathways showed L96 may regulate Th17 cell differentiation, JAK−STAT, and NF−kappa B signaling pathways, which are highly related to tumor development (Supplementary Figure S6). We also conducted GSVA analysis to investigate the pathways related to LY96 across the eight cancers. The top 15 pathways positively and negatively correlated with LY96 are shown in Supplementary Figure S7. The results demonstrated that LY96 was correlated with several immune cell pathways, including CD4, CD8, B cell pathways, and IL-related pathways, including IL2, IL12, and IL17. Otherwise, LY96 was negatively correlated with metabolic-related pathways such as glucose, ether, and cholesterol metabolic processes.

### Correlation Between LY96 and IC50 of Drugs

To explore the resistant properties, we further analyzed the correlation between LY96 expression and IC50 of different types of small molecule drugs using the GDSC2 database (https://www.cancerrxgene.org/). LY96 was positively correlated with the IC50 of dihydrorotenone, TAF1_5496, acetalax, osimertinib, and OF−1 (Suplementary Figure S8) and higher expression of LY96 exhibited higher IC50 in the five types of drugs (Supplementary Figure S8A). Similarly, LY96 was negatively correlated with the IC50 of SB505124, mitoxantrone, irinotecan, topotecan, and fludarabine (Supplementary Figure S8B), higher expression of LY96 often exhibited a lower IC50 value (Supplementary Figure S9).

## Discussion

Our research reveals that the LY96 level is upregulated in most cancers, including STAD, COAD, LIHC, and BRCA, which showed higher expression changes relative to normal tissues. Previous studies obtained similar results in gastric cancer (Zhang et al., 2017), colon cancer (Rajamanickam et al., 2020), and hepatocellular cancer (Qi et al., 2021). However, LY96 was downregulated in breast cancer, and LY96 suppression can inhibit cancer development both *in vivo* and *in vitro* (Zhang et al., 2017; Zheng et al., 2021), which was inconsistent with our result. These conflicting results may be a reflection of patients with a genetic background or molecular type in breast cancer. These results showed LY96 plays an oncogenic role in most cancers.

Next, we found LY96 expression was significantly correlated with TNM stage in some types of cancers, such as BRCA, KIRC, KIRP, BLCA, UVM, MESO, STAD, UVM, SKCM, TGCT, ACC, ESCA, KICH, LIHC, and THCA. LY96 expression was gradually upregulated from TNM I to TNM IV stages in BRCA, KIRC, KIRP, BLCA, UVM, MESO, STAD, and UVM, which indirectly explained the role of LY96 in tumor progression among these cancers. Qi et al. found that suppression LY96 prevents the migration and invasion of hepatocellular carcinoma cells (Qi et al., 2021). Zheng et al. found LY96 silence inhibit migration and invasion of breast cancer cells (Zheng et al., 2021). Furthermore, we analyzed the prognostic role of LY96 across 33 types of cancer. Cox regression analysis indicated LY96 can act as a prognostic indicator related to OS, DSS, and DFI in several cancers, especially acting as an adverse prognostic indicator. Intriguingly, KM analysis yielded these results, and patients with higher LY96 expression obtained shorter survival times than patients with lower LY96 expression.

Tumor copy number variations often predicted the response to immune checkpoint blockade in cancers (Lu et al., 2020) and impacted immune profile and molecular feature (Bassaganyas et al., 2020). Even a new clinical trial revealed that copy number alterations can predict response to neoadjuvant anti-HER2 therapy (Venet et al., 2021). In our analysis, LY96 expression was positively correlated with CNV in UVM, KIRC, LIHC, SKCM, and LUSC; however, there was a negative correlation between LY96 expression and CNV in TGCT, UCEC, STAD, BLCA, COAD, and BRCA. A previous study found that DNA methylation patterns shape the tumor microenvironment and are related to the response of immune treatments in cancers (Mitra et al., 2020). DNA methylation patterns can also alter cell identity and tumor immune surveillance networks (Li et al., 2019). DNA methylation can act as a guide for surveillance and treatment of human cancers (Liang and Weisenberger, 2017). Our results indicated LY96 expression was highly correlated with DNA methylation in different types of cancers. Somatic mutational profiles often shape the tumor microenvironment immune type in most cancers (Koh et al., 2019). Somatic mutation was highly correlated with immune cell infiltration and different immune infiltration patterns (Chakiryan et al., 2021). Our results indicated LY96 expression was highly correlated with the somatic mutation profile in different types of cancers. Microsatellite instability is the result of functional defects in mismatch repair proteins and is also well characterized in other gastrointestinal tumors such as colorectal cancer and gastric cancer. In the latter, higher levels of MSI were associated with better outcomes and increased benefits of immunotherapy (Ghidini et al., 2020). Similarly, tumor mutational load predicts survival after immunotherapy across multiple cancer types (Samstein et al., 2019). Our article demonstrated that LY96 expression was positively correlated with MSI in LAML; however, LY96 expression was negatively correlated with TGCT, CHOL, LUSC, LUAD, and STAD. In terms of TMB, LY96 was positively correlated with TMB in COAD and UCEC, and UCEC was negatively correlated with TMB in CHOL, TGCT, LUAD, STAD, and SKCM. All these results demonstrated that LY96 was correlated with immune-related indicators in most cancers, which may lead to diverse response to immune therapy.

The tumor microenvironment always related to diverse response to immunotherapy and different clinical outcome across cancers (Lei et al., 2020). LY96 was positively correlated with immune, stromal, and estimate scores and was negatively correlated with tumor purity across 33 cancers using the ESTIMATE algorithm. Tumor immune-infiltrated cells in TME are often associated with tumor occurrence and regression across most types of cancer. Our analysis demonstrated that LY96 was positively correlated with tumor-promoting immune cells, including M1 macrophages and T cell regulatory Tregs, whereas high LY96 expression was negatively correlated with tumor immune suppression cells, including activated CD4 memory T cells and CD8 T cells. A study found porphyrin bound to LY96 can suppress immune cell activation (Wang et al., 2020b). Another study found that suppression of LY96 reduced the pro-inflammatory macrophage infiltration (Gibier et al., 2021). These results further indicated that LY96 was correlated with tumor immunity and targeting LY96 may be a promising strategy to develop an antitumor drug.

Lastly, we found that LY96 expression was related to immune-related pathways in GO enrichment analysis and related to JAK−STAT and NF-kappa B signaling pathways in KEGG analysis. Previous studies have detected the role of these pathways across diverse types of cancer. Jiang et al. found that lncRNA RP11-468E2.5 targeting JAK−STAT inhibited colorectal cancer cell proliferation and promoted cell apoptosis (Jiang et al., 2019). Mohrherr et al. found that JAK–STAT inhibition impairs K-RAS-driven lung adenocarcinoma progression (Mohrherr et al., 2019). Another study demonstrated that JAK−STAT linked inflammation to cancer, tumor progression, and even drug resistance (Sabaawy et al., 2021). Qin et al. found that tumor-associated fibroblast-derived IL-6 promotes head and neck cancer progression by the NF-kappa B pathway (Qin et al., 2018). Otherwise, NF-kappa B activation could induce MHC-I antigen presentation to potentiate cancer chemoimmunotherapy (Zhou et al., 2021). Another study showed inhibition of NF-κB c-Rel as a viable therapeutic approach for enhancing checkpoint-targeting immunotherapy protocols (Grinberg-Bleyer et al., 2017). Otherwise, we found that LY96 expression was highly correlated with the IC50 of several drugs, which demonstrated that LY96 plays a part in tumor resistance of some anticancer drugs.

In conclusion, our study first analyzed the pan-cancer role of LY96 across 33 types of cancer. We found that LY96 was differently expressed between tumor and normal tissues and was significantly upregulated in most types of cancers. LY96 was gradually upregulated from stages I to IV in several cancers. Moreover, we found LY96 may play a prognostic role in diverse cancers, and patients with high and low expression often show different clinical outcomes. LY96 was also associated with copy number, DNA methylation, somatic mutation, MSI, TMB, TME characteristics, and immune cell infiltration in cancers. LY96 may also regulate classic tumor-associated pathways in several cancers and is related to drug resistance. This article may help to elucidate the role of LY96 in tumor occurrence and progression, which may promote the development of immunotherapy and targeted therapy in cancers.

## Data Availability

Publicly available datasets were analyzed in this study. These data can be found at: https://portal.gdc.cancer.gov/.
