# Supplementary figures and images for: Pan-Cancer Analysis of the Characteristics of LY96 in Prognosis and Immunotherapy Across Human Cancer
Source: Front Mol Biosci. 2022 May 11;9:837393. doi: 10.3389/fmolb.2022.837393 (PMC9130738; doi:10.3389/fmolb.2022.837393)

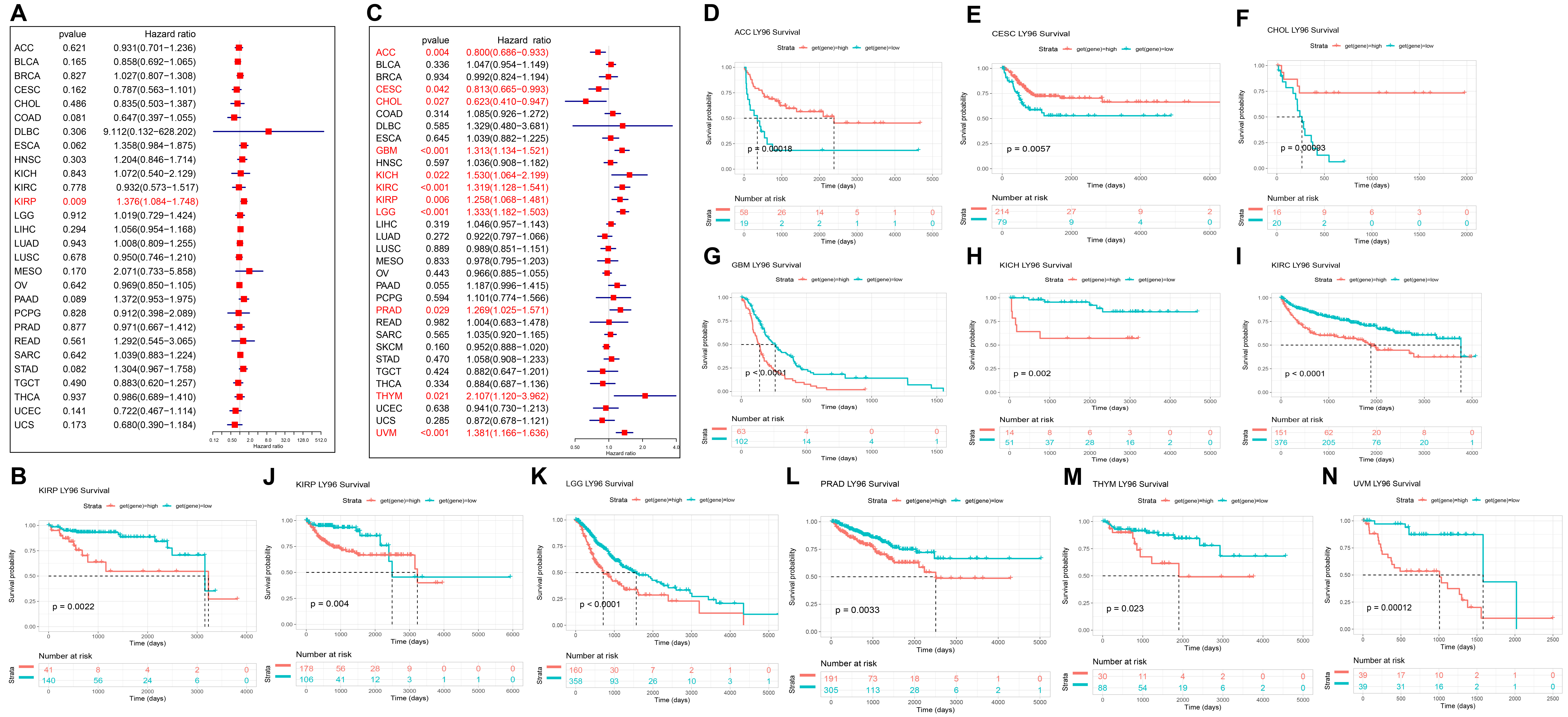

Supplement: Supplementary file 1 [file Image3.JPEG]

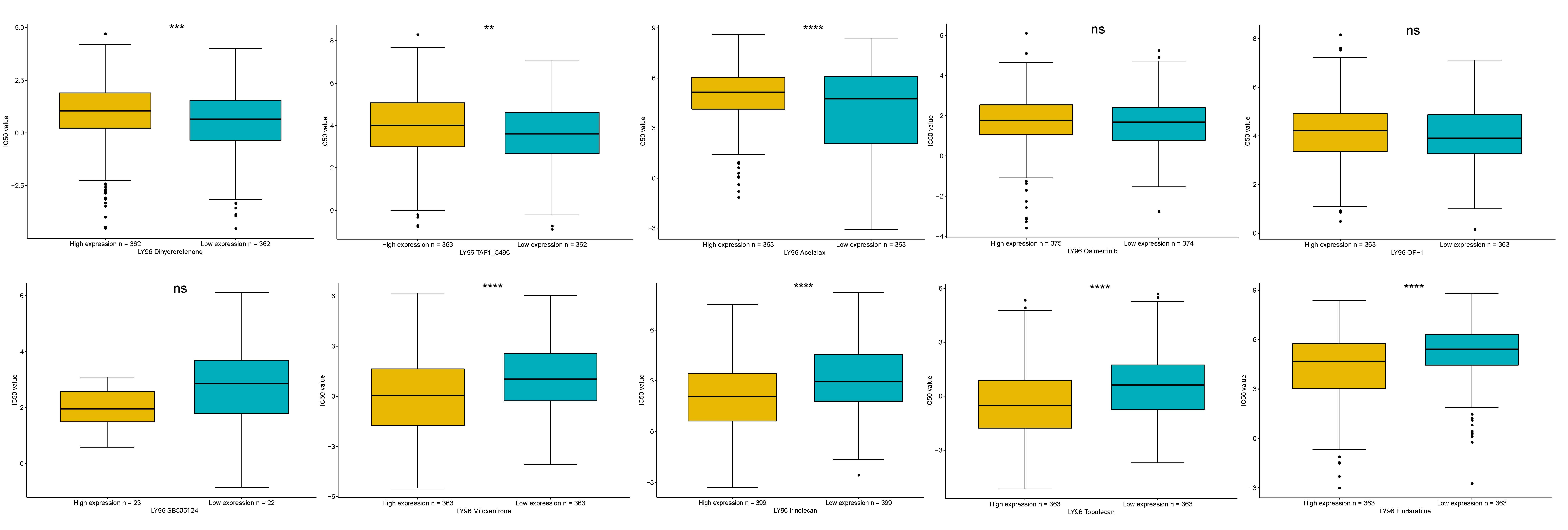

Supplement: Supplementary file 3 [file Image9.JPEG]

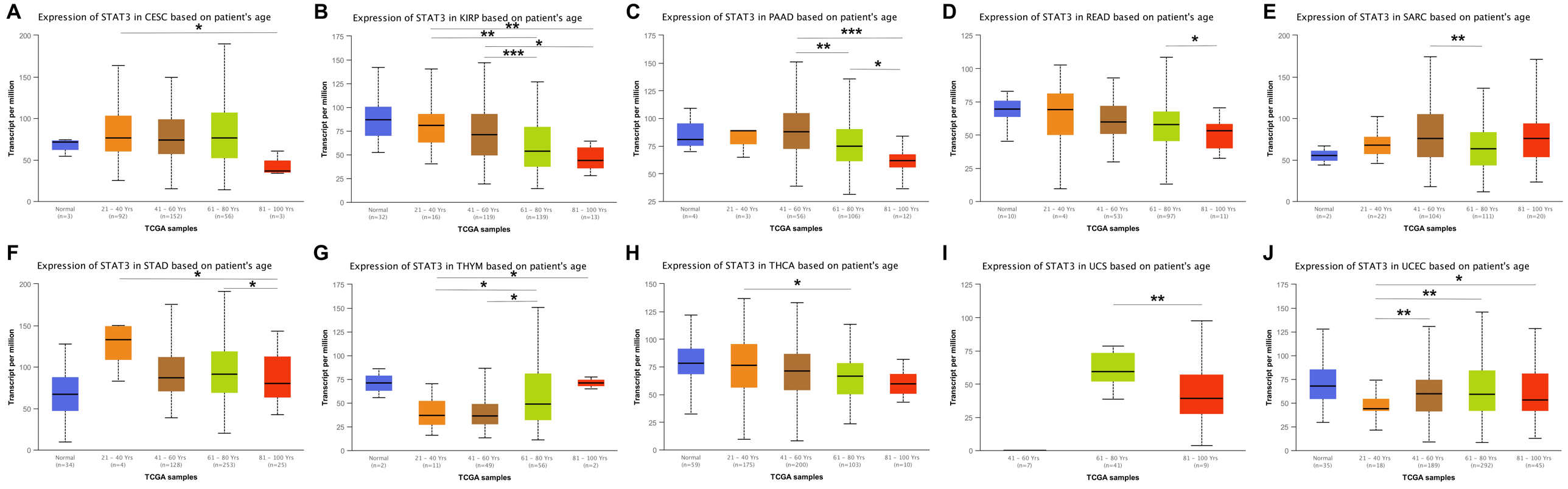

Supplement: Supplementary file 4 [file Image1.JPEG]

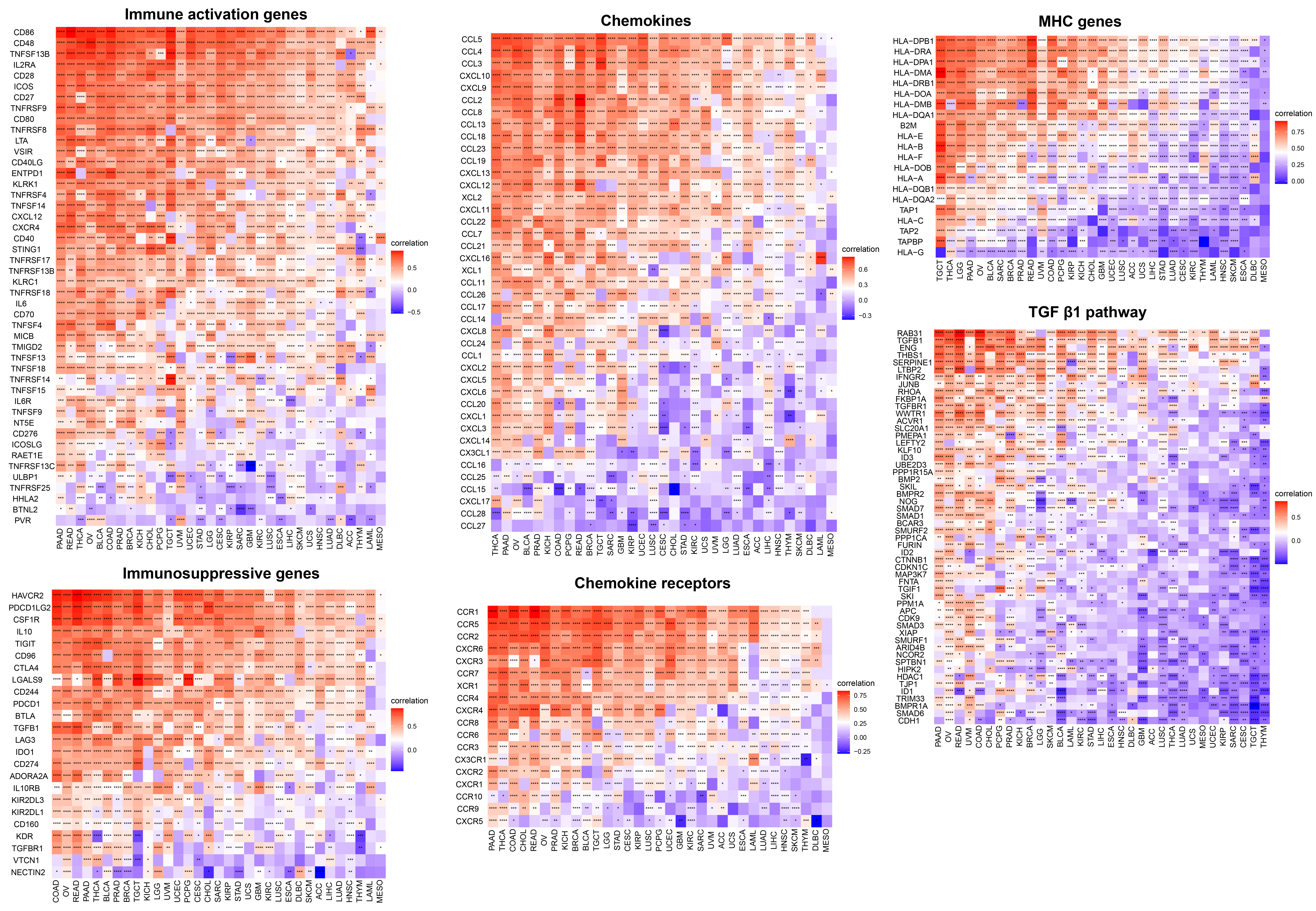

Supplement: Supplementary file 5 [file Image4.JPEG]

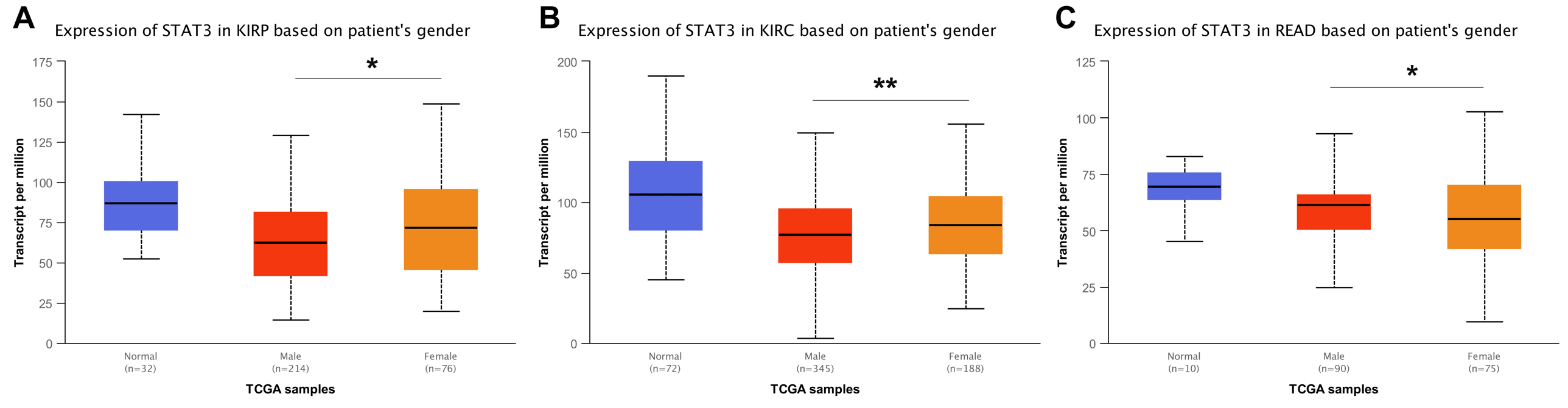

Supplement: Supplementary file 7 [file Image2.JPEG]

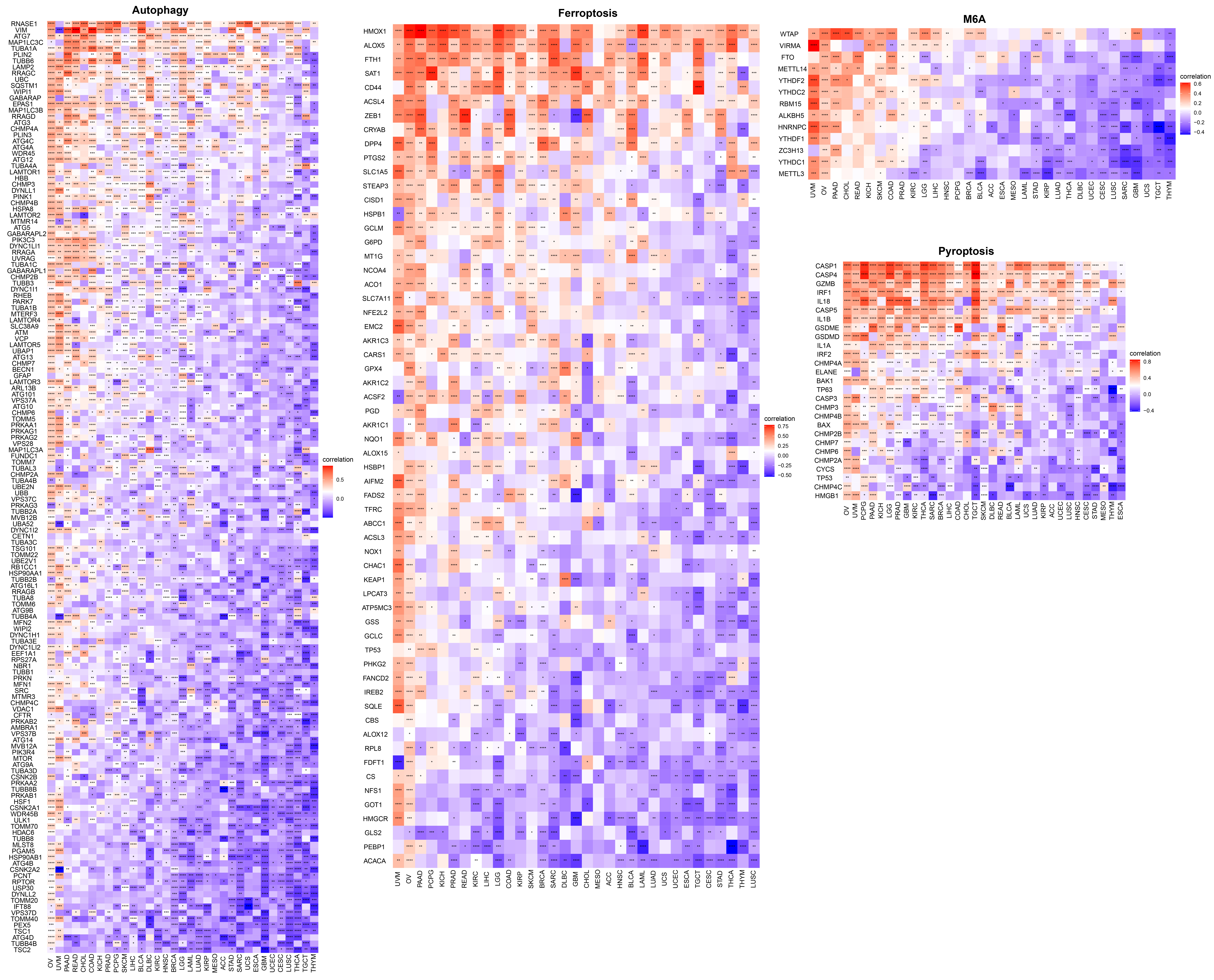

Supplement: Supplementary file 8 [file Image5.JPEG]

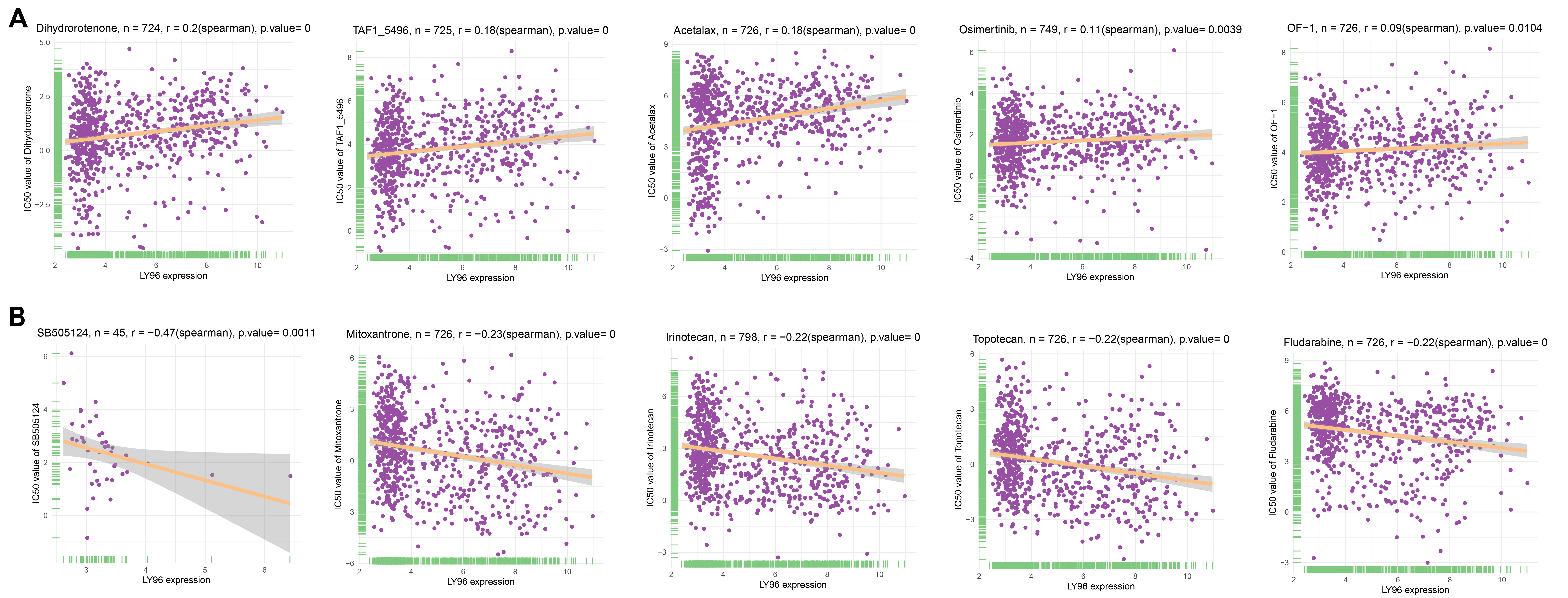

Supplement: Supplementary file 10 [file Image8.JPEG]

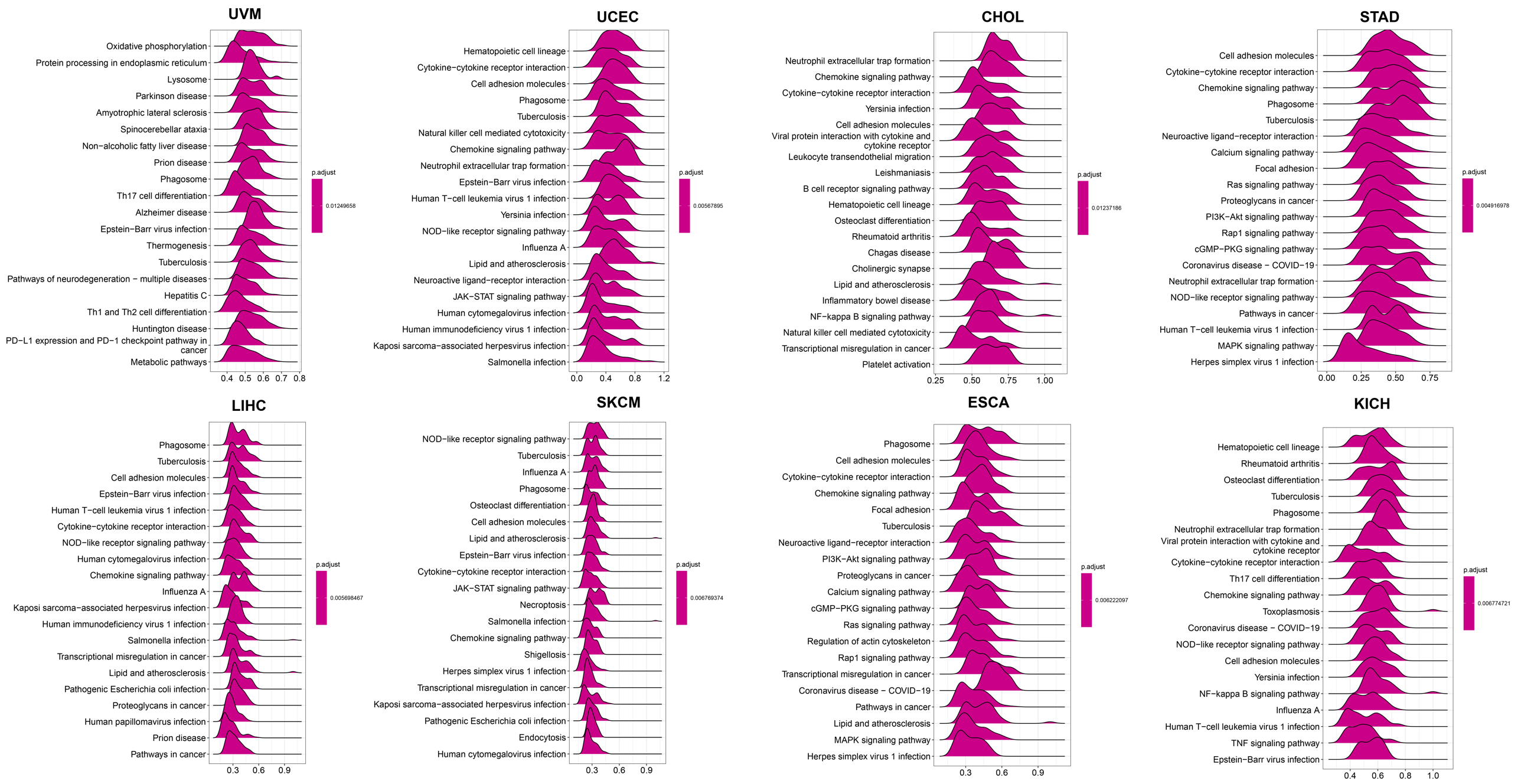

Supplement: Supplementary file 11 [file Image6.JPEG]
