# Supplementary material for: Pan-Cancer Analysis of the Characteristics of LY96 in Prognosis and Immunotherapy Across Human Cancer
Source: Front Mol Biosci. 2022 May 11;9:837393. doi: 10.3389/fmolb.2022.837393 (PMC9130738; doi:10.3389/fmolb.2022.837393)
Supplement: Supplementary file 2 [file Table1.DOCX]

**Table S1. The number of patients of each cancer and their details in our study.**

| Tumor type | Sample size of  tumor tissues from TCGA | Sample size of  normal tissues from TCGA | Sample size of normal tissues from GTEx |
| --- | --- | --- | --- |
| ACC | 77 | 0 | 128 |
| BLCA | 407 | 19 | 9 |
| BRCA | 1098 | 113 | 179 |
| CESC | 306 | 3 | 10 |
| CHOL | 36 | 9 | 0 |
| COAD | 288 | 41 | 308 |
| DLBC | 47 | 0 | 444 |
| ESCA | 182 | 13 | 653 |
| GBM | 165 | 0 | 1152 |
| HNSC | 520 | 44 | 0 |
| KICH | 66 | 25 | 28 |
| KIRC | 531 | 72 | 28 |
| KIRP | 289 | 32 | 28 |
| LAML | 173 | 0 | 70 |
| LGG | 522 | 0 | 1152 |
| LIHC | 371 | 50 | 110 |
| LUAD | 515 | 59 | 288 |
| LUSC | 498 | 50 | 288 |
| MESO | 87 | 0 | 0 |
| OV | 427 | 0 | 88 |
| PAAD | 179 | 4 | 167 |
| PCPG | 182 | 3 | 0 |
| PRAD | 496 | 52 | 100 |
| READ | 92 | 10 | 308 |
| SARC | 262 | 2 | 0 |
| SKCM | 469 | 1 | 812 |
| STAD | 414 | 36 | 174 |
| TGCT | 137 | 0 | 165 |
| THCA | 512 | 59 | 279 |
| THYM | 119 | 2 | 444 |
| UCEC | 181 | 13 | 89 |
| UCS | 57 | 0 | 89 |
| UVM | 79 | 0 | 0 |
