# Supplementary material for: Pan-Cancer Analysis of the Characteristics of LY96 in Prognosis and Immunotherapy Across Human Cancer
Source: Front Mol Biosci. 2022 May 11;9:837393. doi: 10.3389/fmolb.2022.837393 (PMC9130738; doi:10.3389/fmolb.2022.837393)
Supplement: Supplementary file 9 [file Table2.DOCX]

**Table S2. Correlation between LY96 and immune infiltrated cells in BRCA (N=23), THCA(N=20), CESC(N=18), KIRP(N=18) and COAD(N=17).**

| Cell types | BRCA | | THCA | | CESC | | KIRP | | COAD | |
| --- | --- | --- | --- | --- | --- | --- | --- | --- | --- | --- |
|  | correlation | pvalue | correlation | pvalue | correlation | pvalue | correlation | pvalue | correlation | pvalue |
| B.Cells.Memory | 0.081 | 0.008 | 0.159 | 0.000 | 0.203 | 0.000 | 0.262 | 0.000 | 0.146 | 0.014 |
| B.Cells.Naive | 0.151 | 0.000 | 0.316 | 0.000 | 0.043 | 0.454 | 0.178 | 0.003 | 0.103 | 0.085 |
| Dendritic.Cells.Activated | 0.134 | 0.000 | 0.157 | 0.000 | 0.289 | 0.000 | 0.239 | 0.000 | 0.231 | 0.000 |
| Dendritic.Cells.Resting | 0.097 | 0.001 | 0.215 | 0.000 | 0.176 | 0.002 | 0.066 | 0.274 | 0.062 | 0.300 |
| Eosinophils | 0.076 | 0.012 | 0.214 | 0.000 | 0.115 | 0.047 | 0.049 | 0.415 | 0.027 | 0.655 |
| Macrophages.M0 | 0.120 | 0.000 | 0.007 | 0.877 | 0.177 | 0.002 | 0.184 | 0.002 | 0.032 | 0.591 |
| Macrophages.M1 | 0.268 | 0.000 | 0.445 | 0.000 | 0.380 | 0.000 | 0.253 | 0.000 | 0.286 | 0.000 |
| Macrophages.M2 | 0.090 | 0.003 | 0.039 | 0.377 | 0.248 | 0.000 | 0.373 | 0.000 | 0.473 | 0.000 |
| Mast.Cells.Activated | 0.014 | 0.647 | 0.089 | 0.045 | 0.240 | 0.000 | 0.028 | 0.642 | 0.246 | 0.000 |
| Mast.Cells.Resting | 0.150 | 0.000 | 0.352 | 0.000 | 0.048 | 0.403 | 0.426 | 0.000 | 0.296 | 0.000 |
| Monocytes | 0.069 | 0.023 | 0.299 | 0.000 | 0.095 | 0.098 | 0.147 | 0.014 | 0.069 | 0.251 |
| Neutrophils | 0.066 | 0.031 | 0.036 | 0.423 | 0.115 | 0.045 | 0.017 | 0.784 | 0.142 | 0.018 |
| NK.Cells.Activated | 0.017 | 0.584 | 0.093 | 0.036 | 0.097 | 0.094 | 0.132 | 0.028 | 0.054 | 0.370 |
| NK.Cells.Resting | 0.158 | 0.000 | 0.434 | 0.000 | 0.180 | 0.002 | 0.254 | 0.000 | 0.238 | 0.000 |
| Plasma.Cells | 0.092 | 0.002 | 0.302 | 0.000 | 0.185 | 0.001 | 0.015 | 0.806 | 0.254 | 0.000 |
| T.Cells.CD4.Memory.Activated | 0.190 | 0.000 | 0.087 | 0.050 | 0.262 | 0.000 | 0.040 | 0.502 | 0.128 | 0.032 |
| T.Cells.CD4.Memory.Resting | 0.242 | 0.000 | 0.034 | 0.441 | 0.076 | 0.189 | 0.417 | 0.000 | 0.139 | 0.020 |
| T.Cells.CD4.Naive | 0.157 | 0.000 | 0.277 | 0.000 | 0.161 | 0.005 | 0.251 | 0.000 | 0.181 | 0.002 |
| T.Cells.CD8 | 0.102 | 0.001 | 0.112 | 0.012 | 0.189 | 0.001 | 0.130 | 0.030 | 0.162 | 0.007 |
| T.Cells.Follicular.Helper | 0.097 | 0.001 | 0.208 | 0.000 | 0.053 | 0.361 | 0.036 | 0.552 | 0.106 | 0.076 |
| T.Cells.gamma.delta | 0.182 | 0.000 | 0.192 | 0.000 | 0.005 | 0.931 | 0.150 | 0.012 | 0.053 | 0.380 |
| T.Cells.Regulatory.Tregs | 0.201 | 0.000 | 0.355 | 0.000 | 0.189 | 0.001 | 0.250 | 0.000 | 0.183 | 0.002 |
| Lymphocytes | 0.171 | 0.000 | 0.183 | 0.000 | 0.000 | 1.000 | 0.209 | 0.000 | 0.337 | 0.000 |
| Mast.Cells | 0.149 | 0.000 | 0.379 | 0.000 | 0.224 | 0.000 | 0.424 | 0.000 | 0.171 | 0.004 |
| Dendritic.Cells | 0.005 | 0.880 | 0.135 | 0.002 | 0.172 | 0.003 | 0.070 | 0.243 | 0.099 | 0.096 |
| Macrophages | 0.106 | 0.000 | 0.046 | 0.298 | 0.195 | 0.001 | 0.433 | 0.000 | 0.425 | 0.000 |
